# Genome-Wide Association Scan for Variants Associated with Early-Onset Prostate Cancer

**DOI:** 10.1371/journal.pone.0093436

**Published:** 2014-04-16

**Authors:** Ethan M. Lange, Anna M. Johnson, Yunfei Wang, Kimberly A. Zuhlke, Yurong Lu, Jessica V. Ribado, Gregory R. Keele, Jin Li, Qing Duan, Ge Li, Zhengrong Gao, Yun Li, Jianfeng Xu, William B. Isaacs, Siqun Zheng, Kathleen A. Cooney

**Affiliations:** 1 Department of Genetics, University of North Carolina, Chapel Hill, North Carolina, United States of America; 2 Department of Biostatistics, University of North Carolina, Chapel Hill, North Carolina, United States of America; 3 Department of Internal Medicine, University of Michigan, Ann Arbor, Michigan, United States of America; 4 Department of Urology, University of Michigan, Ann Arbor, Michigan, United States of America; 5 Center for Genomics and Personalized Medicine Research, Wake Forest University, Winston-Salem, North Carolina, United States of America; 6 Department of Urology, Johns Hopkins University, Baltimore, Maryland, United States of America; Ohio State University Medical Center, United States of America

## Abstract

Prostate cancer is the most common non-skin cancer and the second leading cause of cancer related mortality for men in the United States. There is strong empirical and epidemiological evidence supporting a stronger role of genetics in early-onset prostate cancer. We performed a genome-wide association scan for early-onset prostate cancer. Novel aspects of this study include the focus on early-onset disease (defined as men with prostate cancer diagnosed before age 56 years) and use of publically available control genotype data from previous genome-wide association studies. We found genome-wide significant (p<5×10^−8^) evidence for variants at 8q24 and 11p15 and strong supportive evidence for a number of previously reported loci. We found little evidence for individual or systematic inflated association findings resulting from using public controls, demonstrating the utility of using public control data in large-scale genetic association studies of common variants. Taken together, these results demonstrate the importance of established common genetic variants for early-onset prostate cancer and the power of including early-onset prostate cancer cases in genetic association studies.

## Introduction

Prostate cancer (PCa) is a leading cause of cancer mortality in men. In 2013, it is estimated that 238,590 men will be diagnosed with and 29,720 men will die from the disease [1]. Approximately 1 in 6 men will be diagnosed with PCa during their lives based on the current incidence rates [1], [2]. The major recognized risk factors for PCa are increasing age, African ancestry and positive family history.

Genome-wide association (GWA) studies and follow-up studies have identified and replicated ∼65 single-nucleotide polymorphisms (SNPs) that are associated with PCa in men of European descent [3]–[17]. Most of these studies have included primarily older PCa cases, reflecting the demographics of the disease as well as, in some cases, study design constraints. For most complex disorders, including common cancers, early age at diagnosis is a marker of heritable forms of the disease. Among hereditary PCa families, disease is diagnosed 6–7 years younger than sporadic disease and the risk for PCa increases with decreasing age of affected family members [18]. Further, studies have suggested that men diagnosed with PCa earlier in life are more likely to die from their disease compared to men, with similar clinical features of disease, diagnosed at an older age [19], [20]. To assess the importance of common genetic variants to early-onset PCa, we performed a GWA study for early-onset PCa, defined here as PCa diagnosed prior to age 56 years, in 931 men of European descent who were diagnosed with PCa at an average age of 49.7 years and 4120 European descent controls. This study represents the largest GWA study to date focusing specifically on men with early-onset PCa.

## Materials and Methods

### Ethics Statement

The University of Michigan IRBMED has reviewed and approved the scheduled continuing review (SCR) submitted for the University of Michigan Prostate Cancer Genetics Project. The IRB determined that the proposed research continues to conform with applicable guidelines, State and federal regulations, and the University of Michigan's Federal-wide Assurance (FWA) with the Department of Health and Human Services (HHS). All University of Michigan subjects included in this study provided written informed consent to participate in the study; the protocol and consent documents were approved by the Institutional Review Board at the University of Michigan Medical School.

Genotype data from follow-up samples for this study were obtained from Johns Hopkins University (JHU). This human subjects research proposal was reviewed and approved by the Johns Hopkins Medicine Institutional Review Board (JHM IRB). JHU PCa case DNA were obtained from de-identified pathological specimens and determined, by JHM IRB, to be exempt from the requirement of written or oral consent. Follow-up control DNA samples were obtained from PCa screened men negative for the disease. All JHU controls provided written informed consent; the protocol and consent documents were approved by JHM IRB. Analyses for this study were conducted at the University of North Carolina at Chapel Hill using de-identified data. The University of North Carolina Institutional Review Board approved the proposed study. Data material transfer agreements were signed between officials at the University of North Carolina, University of Michigan and Johns Hopkins University.

### Study Samples

The final study case sample included 931 successfully genotyped unrelated early-onset PCa cases (diagnosed prior to age 56 years) of European descent from the University of Michigan Prostate Cancer Genetics Project (UM-PCGP). Descriptive information about the cases is presented in Table 1. The average (standard deviation) and median age (range) of prostate cancer diagnosis in these 931 cases was 49.7 (4.1) years and 50 (27–55) years, respectively. Of note, this sample of men is enriched for positive family history (576/931 or 61.9% with reported first or second degree relatives with PCa), partially a consequence of some samples (n = 127) being ascertained from families included in the UM-PCGP linkage study on hereditary PCa. Descriptions of the UM-PCGP hereditary PCa families can be found elsewhere [21], [22]. A total of 351 cases came from families that had DNA collected on multiple cases; 817/931 cases were either family probands or ascertained directly due to early age at diagnosis. In families that had more than one PCa case diagnosed prior to age 56 years, only the youngest available case was included in the current study. Clinical features of UM-PCGP early-onset PCa cases are presented in Table 1.

**Table 1:** Characteristics of 931 UM-PCGP Early-Onset Prostate Cancer Cases^1^.

Clinical Trait	Mean (Standard Deviation)	Median (Range)
Age at Diagnosis (years)	49.7 (4.1)	50 (27–55)
Prediagnostic PSA (mg/dL)^2^	20.6 (199.5)	5.2 (0.4–5428)
Gleason Score	N^3^	%
≤6	410	44.6
7	427	46.4
≥8	83	9.0
T Stage	N^4^	%
T1	1	0.1
T2	660	82.1
T3	140	17.4
T4	3	0.4

1 Includes 20 metastatic cases and 32 cases with lymph node involvement.

2 Prediagnostic PSA available on 870 cases.

3 Gleason scores available on 920 cases. Note: Prostatectomy Gleason used when available (n = 787), otherwise biopsy Gleason scores used (n = 133).

4 T Stage available on 804 cases.

Unrelated controls with GWA study SNP data were selected from publically available resources through dbGap (www.ncbi.nlm.nih.gov/gap) and Illumina (www.illumina.com). Controls were selected to have European reported ancestry and genotype data generated from a GWA study commercial platform similar to the platform used in UM-PCGP cases. To maintain independent results from prior published PCa GWA studies, public controls that were used in these prior PCa studies were excluded from consideration. Controls, which included women, were not, to our knowledge, screened for PCa. Controls came from the Cancer Genetics Markers of Susceptibility (CGEMS) (n = 1135) GWA study for breast cancer [23] and Illumina's iControlDB database (n = 2985) (www.Illumina.com). Only CGEMS breast cancer *controls* were included. Limited descriptive information, including age, gender and ancestry, on selected iControlDB subjects can be obtained from the Illumina website. The rationale for including female controls is provided in the Discussion. Separate analyses including only male iControlDB subjects were also performed.

A subset of novel SNPs (p<5.0×10^−5^ and not previously reported to be associated with PCa) were analyzed in an additional sample of 2571 unrelated PCa cases (1053 diagnosed prior to age 56 years) and 921 screened controls of European-descent from JHU (see Ewing et al. [24] for description of subjects).

### Genotyping

938 European-American UM-PCGP early-onset PCa cases were initially genotyped at Wake Forest University using Illumina's HumanHap 660W-Quad v1.1 BeadChip. CGEMS Breast cancer controls were genotyped previously using Illumina's HumanHap550v1 [23]. The iControlsDB subjects were genotyped previously using Illumina's HumanHap550v1 (n = 1478) or HumanHap550v3 (n = 1507) commercial genotyping platforms. Follow-up genotyping on JHU subjects was performed at Wake Forest University using the Sequenom system. All the procedures followed the manufacturer's iPLEX Application Guide (Sequenom, Inc. SanDiego, CA) and all the assay reagents were purchased from Sequenom. To ensure the quality of the genotyping, around 2% of the sample duplicates and 2% of the negative controls, in which water was substituted for DNAs, were applied.

### Statistical Analyses

Genotyping quality control (QC) methodology was uniformly applied to all samples. To reduce the possible impact of bias due to “batch” genotyping effects, SNPs missing genotype calls in >2% of subjects in *any* of the four sample sets (UM-PCGP cases, CGEMS breast cancer controls, Illumina iControls V1 or iControls V3) were excluded. Subjects missing >5% of SNP genotyping calls were also excluded. For UM-PCGP cases, genotyping calls between Illumina's HumanHap 660W-Quad v1.1 BeadChip results and 14 SNPs previously genotyped using TaqMan [25] were compared to verify sample identity and to assess the overall concordance of genotype calls between the two platforms. In addition, 21 duplicate samples were included to assess concordance of genotype calls with the Illumina's HumanHap 660W-Quad v1.1 BeadChip results. Laboratory personnel were blinded to the identity of the duplicates. European ancestry for all subjects, including controls, was verified using the software ADMIXTURE [26]; subjects with apparent misidentified ancestry or mixed ancestry were removed from consideration.

Genotype imputation was performed to expand the coverage of variants in our GWA study to SNPs that were not included on Illumina's HumanHap 660W-Quad v1.1 BeadChip or that were included on the BeadChip but were lost during QC, using the software package MaCH [27], [28]. Genotype imputation was performed separately including SNPs from HapMap Phase II (CEU reference samples) and HapMap Phase III (CEU+TSI reference samples). Imputed genotype data were analyzed as dosage values (expected number of copies of the minor alleles) in logistic regression models implemented in Mach2dat [28]. The logistic regression models included covariate adjustment for the first 10 principal components for ancestry and/or batch effects. Principal component analysis was performed using the software Eigenstrat [29] on the combined sample of cases and controls using a linkage-disequilibrium (LD) pruned set of SNPs. All genotype data for SNPs that were excluded based on quality control analyses due to genotype-missing rates in one or more of the four sample sets were zeroed out in all four target sample sets prior to imputation to reduce the possibility of batch genotype effects impacting the imputation-based SNP association results. Preference was given to Phase III imputation results when a SNP was successfully imputed using both Phase II and Phase III HapMap samples. Genome-wide significance was defined as p<5.0×10^−8^. Chromosome X variants were not imputed.

Single variant association analyses for directly genotyped SNP data were also performed using the software PLINK [30]. Logistic regression models were systematically analyzed with covariate adjustment for the first 10 principal components derived from Eigenstrat. Only SNPs that were genotyped >98% rate in all four sets of samples were included in the genotyped-SNP analyses. Chromosome X analyses were performed on directly genotyped SNPs and limited to include only the 1126 male iControlDB subjects.

A subset of SNPs reaching p<5×10^−5^ in the GWA study were followed up in an independent sample of 2571 PCa cases and 921 screened controls from JHU. SNPs were analyzed individually using chi-square tests. Subset analyses were performed restricting cases to those (n = 1053) diagnosed with PCa prior to age 56 years.

## Results

592,652 SNPs were genotyped on 938 unrelated European-American UM-PCGP cases with early-onset PCa. QC analyses were conducted to assess overall accuracy and completeness of genotype data. Five UM-PCGP subjects were removed for low genotype rate (<95% of SNPs with genotype data). Two additional UM-PCGP subjects had large estimated proportions of non-European ancestry and were removed. After sample removal, a total of 931 unrelated UM-PCGP PCa cases passed QC and were included in the study. Genotype concordance rates between HumanHap 660W-Quad v1.1 BeadChip and Taqman genotype calls was >99% and internal concordance of HumanHap 660W-Quad v1.1 BeadChip calls in 21 duplicate pairs was >99.99%.

**Figure 1:**
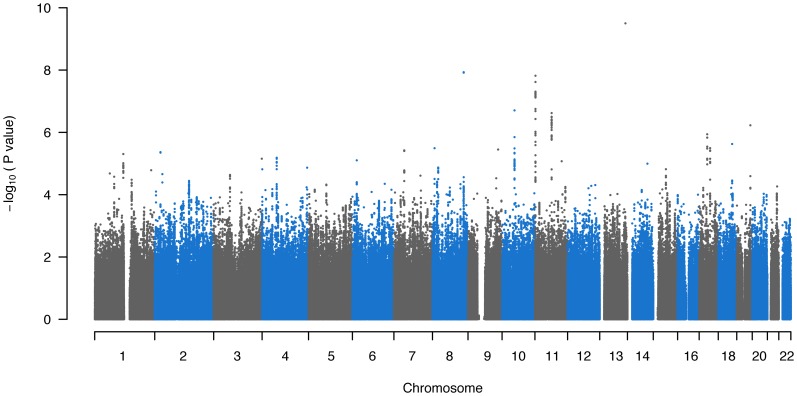
Manhattan Plot of Results for Imputed HapMap Phase II and Phase III SNPs.

A total of 458,162 autosomal SNPs with a successful genotyping rate >98% in each sample (UM-PCGP, CGEMS breast cancer controls, iControls V1, iControls V3) were included in the final target set for genotype imputation. Genotype imputation allowed a total of 2,639,562 autosomal SNPs, with MaCH imputation quality score R^2^ >0.3, to be analyzed for association with PCa. Results across the genome are graphically illustrated in Figure 1 and the top findings (p<1.0×10^−5^) are presented in Table 2. The top result was for an uncommon (minor allele frequency estimated to be 1.5% in combined case-control sample) chromosome 13 SNP rs11839053 (p = 8.7×10^−10^) based on HapMap Phase II imputation data. For reasons described in the Discussion, we believe the result for this SNP should be considered with caution. Two established 8q24 SNPs (rs10505477, p = 9.4×10^−9^; rs6983267, p = 1.2×10^−8^) and two established 11p15 SNPs (rs7126629, p = 2.3×10^−8^; rs7114836, p = 3.7×10^−8^) also reached genome-wide significance. The top novel results were for Chromosome 18 SNP rs11664910 (p = 2.3×10^−6^) and Chromosome 17q21-22 SNP rs8064701 (p = 4.8×10^−6^).

**Table 2:** Summary of top GWAS results (p<1.0×10^−5^).

Chr.	Num SNPs p<10^−5^	SNP with lowest p-value	Location	Allele (1/2)	Freq Allele 1 Case/Control	R^2^	OR^1^	SE	P-Value	Nearest Gene	Novel/Previously Reported
1	28	rs7514323	117804242	A/G	0.18/0.23	0.92	0.73	0.069	3.2×10^−6^	Intron *MAN1A2*	Novel
2	2	rs4665609	23628257	A/C	0.50/0.44	1.00	1.28	0.053	4.2×10^−6^	5′ *UBXD4*	Novel
3	1	rs6789365	198039349	A/C	0.24/0.17	0.38	1.61	0.11	9.3×10^−6^	Intron *PAK2*	Novel
4	12	rs1486307	60123317	A/G	0.24/0.29	0.96	0.76	0.062	5.0×10^−6^	5′ *LPHN3*	Novel
6	5	rs3734234	17902473	A/G	0.59/0.65	0.99	0.79	0.053	7.0×10^−6^	Syn. *KIF13A*	Novel
7	15	rs1880408	42574162	G/A	0.09/0.05	0.98	1.63	0.11	7.1×10^−6^	5′ *GLI3*	Novel
8	3	rs7013418	8538732	G/T	0.29/0.23	0.96	1.35	0.064	3.7×10^−6^	5′ *CLDN23*	Novel
8	4	rs10505477	128476625	G/A	0.42/0.51	0.97	0.72	0.054	9.4×10^−9^	5′ *MYC*	Reported
9	1	rs16911551	123990298	T/C	0.10/0.05	0.46	1.97	0.15	6.2×10^−6^	Intron *C9orf18*	Novel
10	36	rs10993994	51219502	T/C	0.49/0.42	0.99	1.32	0.054	1.9×10^−7^	5′ *MSMB*	Reported
11	41	rs7126629	2185528	C/A	0.27/0.21	0.86	1.44	0.064	2.3×10^−8^	5′ *TH*	Reported
11	30	rs11228583	68765690	G/T	0.41/0.48	0.97	0.75	0.055	2.0×10^−7^	5′ *MYEOV*	Reported
13	1	rs11839053	105861043	C/T	0.05/0.01	0.65	4.02	0.22	8.7×10^−10^	3′ *EFNB2*	Novel
14	1	rs2150333	82240475	T/A	0.04/0.08	0.62	0.50	0.16	2.9×10^−6^	3′ *SEL1L*	Novel
17	8	rs2005705	33170413	A/G	0.37/0.44	0.73	0.74	0.063	9.6×10^−7^	Intron *TCF2*	Reported
17	8	rs8064701	45144864	G/C	0.15/0.11	0.99	1.43	0.076	4.8×10^−6^	Intron *FAM117A*	Novel
18	4	rs11664910	57179043	G/A	0.46/0.37	0.78	1.42	0.062	2.3×10^−6^	5′ *CDH20*	Novel
19	4	rs17632542	56053569	C/T	0.04/0.08	0.95	0.54	0.12	1.0×10^−7^	Missense *KLK3*	Reported

1 Allele 1 (minor allele) is effect/risk allele.

Results for analyses of directly genotyped SNPs were consistent with results from the imputed genotype data for SNPs included in both datasets (data not shown). Of note, rs6983267 also reached genome-wide significance in the genotyped-SNP analyses (p = 1.3×10^−8^). Little evidence for a systematic inflated type I error was observed when taking into account the distribution of all results (genomic inflation factor 1.026) [31]. A total of 11,397 directly genotyped SNPs on chromosome X were also analyzed; the top finding was located at rs5906300 (p = 8.1×10^−5^) and there was no evidence for any systematic inflation of type I error across the X chromosome (Genomic inflation factor = 1.00).

Thirty-nine SNPs previously reported to be associated with PCa in men of European descent, summarized in Goh et al. [32], were evaluated for confirmatory evidence in our study of men with early onset disease (Table 3). Twenty-three out of 39 SNPs were at least nominally significant (p<0.05) in the current study; all 23 had directions of effect consistent with the previous reports. Twelve of the 16 SNPs that did not reach nominal significance also had direction of effect consistent with the previous reports. Estimated imputation quality for the vast majority of these SNPs was excellent.

**Table 3:** Results at established PCa loci in men of European descent based on loci presented in Goh et al. [32]. Results presented for imputed SNPs.

Locus	SNP (Rare/Common^1^)	Per allele OR Discovery Study	Per allele OR Current Study	P-value	Imputation Quality (R^2^)
2p11	rs10187424 (G/A)	0.92	0.94	0.27	1.00
2p15	rs721048 (A/G)	1.15	1.17	0.021	1.00
2p21	rs1465618 (A/G)	1.08	1.04	0.57	0.94
2q31	rs12621278 (G/A)	0.75	0.58	5.2×10^−5^	1.00
2q37	rs2292884 (G/A)	1.14	1.08	0.18	1.00
3p12	rs2660753 (T/C)	1.18	1.09	0.29	0.99
3q21	rs10934853 (A/C)	1.12	1.09	0.15	1.00
3q23	rs6763931 (T/C)	1.04	1.10	0.074	1.00
3q26	rs10936632 (C/A)	0.90	0.84	0.0035	0.75
4q22	rs17021918 (T/C)	0.90	0.97	0.59	0.99
4q22	rs12500426 (A/C)	1.08	1.14	0.012	0.99
4q24	rs7679673 (A/C)	0.91	0.88	0.017	0.98
5p12	rs2121875 (G/T)	1.05	0.99	0.84	1.00
5p15	rs2242652 (A/G)	0.87	0.88	0.23	0.49
6p21	rs130067 (G/T)	1.05	0.98	0.80	1.00
6q25	rs9364554 (T/C)	1.17	1.24	1.9×10^−4^	1.00
7p15	rs10486567 (A/G)	0.74	0.83	0.0038	1.00
7q21	rs6465657 (C/T)	1.12	1.17	0.0025	1.00
8p21	rs2928679 (T/C)	1.05	0.96	0.51	1.00
8p21	rs1512268 (A/G)	1.18	1.23	1.2×10^−4^	1.00
8q24	rs1447295 (A/C)	1.62	1.38	7.8×10^−5^	1.00
8q24	rs6983267 (G/T)	1.26	1.36	9.5×10^−9^	1.00
8q24	rs16901979 (A/C)	1.79	1.39	0.010	1.00
8q24	rs10086908 (T/C)	0.87	0.88	0.027	1.00
8q24	rs12543663 (C/A)	1.08	1.25	9.1×10^−5^	1.00
8q24	rs620861 (A/G)	0.90	0.80	8.8×10^−5^	0.97
9q33	rs1571801 (T/G)	1.27	1.06	0.29	0.99
10q11	rs10993994 (T/C)	1.25	1.32	1.9×10^−7^	0.99
10q26	rs4962416 (C/T)	1.20	1.20	0.0014	1.00
11p15	rs7127900 (A/G)	1.22	1.40	1.0×10^−7^	1.00
11q13	rs7931342 (T/G)	0.84	0.77	1.2×10^−6^	1.00
12q13	rs10875943 (C/T)	1.07	1.05	0.37	1.00
12q13	rs902774 (A/G)	1.17	0.99	0.91	1.00
17q12	rs4430796 (A/G)	1.22	1.33	2.5×10^−6^	0.75
17q12	rs11649743 (A/G)	1.28	1.10	0.17	1.00
17q24	rs1859962 (G/T)	1.20	1.21	2.6×10^−4^	1.00
19q13	rs2735839 (A/G)	0.83	0.72	3.7×10^−5^	1.00
22q13	rs5759167 (T/G)	0.86	0.84	0.0014	1.00
Xq12	rs5919432 (G/A)	0.94	0.93	0.56	Genotyped

1 Rare allele is the coded effect/risk allele.

Results from association analyses only including the 1126 male iControlDB subjects were similar to those obtained using the larger sex-combined control sample. Genome-wide significant findings were obtained for the two aforementioned chromosome 8q24 SNPs (rs10505477, p = 1.7×10^−9^; rs6983267, p = 1.8×10^−9^) and known chromosome 17 *TCF2*-intronic SNP rs4430796 (p = 4.1×10^−8^). Chromosome 11p15 SNPs rs7126629 (p = 1.6×10^−6^) and rs7114836 (p = 9.9×10^−6^) and Chromosome 13 SNP rs11839053 (p = 1.2×10^−4^) did not reach genome-wide significance when using the smaller control sample.

Thirteen independent SNPs that demonstrated strong nominal association with PCa (defined here as p<5×10^−5^), when using the complete control sample, and that have not been previously implicated to be associated with PCa were genotyped and tested for association with PCa in an independent sample of 2571 unrelated European-descent PCa cases and 921 screened controls from JHU. When results were similar between the top imputed SNP and a directly genotyped SNP in the same region, the SNP directly genotyped was selected for follow-up. Only one SNP, rs11664910, reached nominal significance (p<0.05); however, the direction of effect for this SNP was not consistent with the initial GWA study result (Table 4). Results were similar when restricting the follow-up case sample to cases diagnosed prior to age 56 years (data not shown).

**Table 4:** Results for 13 SNPs with p<5×10^−5^ in the GWA study in a follow-up study of 2571 PCa cases and 921 screened controls from JHU.

				GWA Study				Follow-Up Study		
Chr	Location	SNP	Alleles (1/2)	Freq Allele 1 Case/Control	OR^1^	p-value	R^2^	Freq Allele 1 Case/Control	OR^1^	p-value
1	117717430	rs1146298	G/A	0.20/0.25	0.76	7.9×10^−6^	1.00	0.24/0.25	0.99	0.93
1	152208138	rs11264743	T/C	0.35/0.30	1.26	4.5×10^−5^	1.00	0.31/0.31	1.02	0.78
2	23628257	rs4665609	A/C	0.50/0.44	1.28	4.2×10^−6^	1.00	0.45/0.46	0.93	0.19
4	60114549	rs10517468	G/T	0.22/0.27	0.77	1.1×10^−5^	1.00	0.25/0.26	0.94	0.30
4	185567394	rs3775554	G/C	0.16/0.11	1.46	1.9×10^−5^	0.80	0.12/0.12	0.96	0.61
6	133071135	rs12527885	C/T	0.04/0.07	0.56	1.5×10^−5^	0.96	0.06/0.05	1.11	0.38
7	42574162	rs1880408	G/A	0.09/0.05	1.63	7.1×10^−6^	0.98	0.06/0.07	0.85	0.15
8	8538732	rs7013418	G/T	0.29/0.23	1.35	3.7×10^−6^	0.96	0.25/0.24	1.06	0.35
9	123990298	rs16911551	T/C	0.10/0.05	1.97	6.2×10^−6^	0.46	0.04/0.03	1.22	0.19
13	105861043	rs11839053	C/T	0.05/0.01	4.02	8.7×10^−10^	0.65	0.04/0.05	0.87	0.29
14	82240475	rs2150333	T/A	0.04/0.08	0.50	2.9×10^−6^	0.62	0.08/0.08	0.99	0.88
17	45153724	rs7225566	T/C	0.15/0.11	1.42	5.8×10^−6^	1.00	0.12/0.11	1.14	0.13
18	57179043	rs11664910	G/A	0.45/0.38	1.34	2.3×10^−6^	0.78	0.36/0.40	0.87	0.01

1 Odds Ratio: Effect/risk allele is allele 1 (minor allele).

## Discussion

From 2005–2009, the average age at PCa diagnosis in the United States was 67 years and only ∼10% of cases were diagnosed prior to age 55 years [1]. Given the small proportion of PCa cases diagnosed in this age range, most genetic studies for PCa are concentrated on men diagnosed with the disease later in life despite the evidence that early age at diagnosis is an indicator of increased genetic susceptibility. For example, a Swedish study has shown that family history is particularly important in men who have one or more first-degree relatives that were diagnosed with PCa at a relatively young age [19]. The relative risk for developing PCa for a man whose father had been diagnosed with PCa at age 60 or older was estimated to be 1.5. The relative risk for developing PCa increased to 2.5 if the father was diagnosed prior to 60 years of age. Similarly, if one brother was diagnosed with PCa at age 60 or older then the relative risk for a man developing PCa was estimated to be 2 whereas the relative risk was estimated to be 3 if that brother was diagnosed with PCa prior to age 60 [19]. In a meta-analysis, the risk of PCa was shown to increase with decreasing age at PCa diagnosis of a first-degree relative [20].

We describe a GWA study for early-onset PCa based entirely of cases diagnosed with the disease prior to age 56 years. A single novel locus, chromosome 13 SNP rs11839053 (p = 8.7×10^−10^), reached genome-wide significance (p<5×10^−8^), though we urge caution in interpreting this result (see below). A total of four variants in known regions of PCa association reached genome-wide significance: two 8q24 variants, rs6983267 (p = 9.5×10^−9^) and rs10505477 (p = 9.4×10^−9^), and two 11p15 variants, rs7126629, (p = 2.3×10^−8^) and rs7114836, (p = 3.7×10^−8^). In addition to these loci, there was strong supportive evidence at a number of previously established PCa loci (Table 3). Of note, for the established loci the observed odds ratios were comparable to the odds ratios in the initial discovery studies despite the likely upwards biased odds ratio estimates in the original reports, due to the “winners curse” phenomenon in SNP association discovery [33], and the use of female and unscreened male controls in the current study.

In this report, we observed one novel significant association for chromosome 13 SNP rs11839053 based on HapMap Phase II imputation data (p = 8.7×10^−10^). We noted a strong discrepancy between results from HapMap Phase II (p = 1.0×10^−9^) and Phase III (p = 0.98) imputation results for neighboring SNP rs11843540, which is in strong LD with rs11839053 (R^2^ = 1.0 in HapMap Phase II CEU samples). Rs11839053 was not genotyped in HapMap Phase III samples. The strong discrepancy between results for rs11843540 based on Phase II and Phase III imputation data was the only noted major difference between these two data sets across all SNPs that were imputed using both reference samples; results were also highly concordant between genotyped and imputed SNPs (Spearman's correlations: 0.98, 0.98, 0.96, between results for Phase II vs. genotype, Phase III vs. genotype, and Phase III vs. Phase II, respectively). Interestingly, the significant result at rs11839053 was also observed when restricting analyses to the CGEMS breast cancer controls and when analyzing imputed genotype data generated using 1000 Genomes Project data (3^rd^ release) as the reference panel (data not shown). We note that imputation qualities for rs11839053 and rs11843540 were relatively poor (r^2^∼0.6 in all reference panels for each SNP), we observed little evidence for association (all p>0.001) for any directly genotyped SNPs in the 500 kb region immediately surrounding the two SNPs, and we did not observe any evidence for association at rs11839053 in our follow-up study of 2571 cases and 921 screened controls from JHU (Table 3). While our study using public controls appeared to have good overall control of type I error, any individual result should be considered suspect. It is unclear whether the result at rs11839053 in our GWA study is an artifact of using public control genotype data (i.e. “batch” effects for one or more genotyped SNPs in the region impacting imputation) or a true signal. Future studies will be necessary to confirm the association result before the locus should be considered a legitimate PCa locus.

We identified 12 additional novel regions that contained variants that had suggestive evidence for association (defined here as p<5×10^−5^). A representative SNP was chosen in each region and followed up in the JHU samples; no significant evidence supporting any of the results in the initial study were observed (Table 3). Arguably the most interesting result among these twelve loci was for chromosome 17q21-22 imputed SNP rs8064701 and nearby directly genotyped SNP rs7225566. Recently we discovered an uncommon missense variant, G84E/rs138213197, in *HOXB13* that is associated with PCa [24]. The G84E variant is ∼1.2 Mb proximal to rs8064701 and rs7225566. Among the 931 cases in the current study (which were also included in the initial *HOXB13* report), 23 (∼2.5%) carried the variant allele at *HOXB13*. We performed long-range haplotyping using FastPhase2 [34] and identified a single long-range haplotype that contained all 23 G84E variant alleles (a single case without the variant allele also was predicted to have the same long-range haplotype). The frequency of the minor (risk) allele for rs7225566 in the GWA study was 15% in cases and 11% in controls. Fifteen of the 23 cases carrying the *HOXB13* G84E risk allele also carried the minor/risk allele for rs7225566, including one homozygote. These results suggest the observed nominally significant associations at rs8064701 and rs7225566 are partially due to linkage disequilibrium with *HOXB13* G84E. While there was a slight increase in frequency of the rs7225566 risk allele in the JHU data (11% in cases versus 10% in controls), the result did not reach statistical significance. Finally, we note that rs7225566 is ∼362 kb distal to rs7210100, an uncommon variant which was previously identified to be associated with PCa in a GWA study of African Americans [35]. Rs7210100 was not directly genotyped or successfully imputed, due to the absence of Caucasian carriers in the HapMap reference panels, in our GWA study samples. The absence/rarity of the risk allele for rs7210100 in populations of European descent strongly suggests our finding at rs7225566 is independent of this previous reported variant. Of note, as previously reported (Supplemental Material of Ewing et al. [24]), among 24 African American rs7210100 risk-allele carriers, none carried the *HOXB13* G84E risk allele.

Our initial discovery study included only publically available control genotype data in contrast to using a gold-standard age-matched screened control sample. The UM-PCGP, being a family-based and case-only study, does not have access to an ideal large control sample from the same population as the cases. Disease misclassification, which would likely occur at higher rates when using public control data, can cause a reduction in statistical power to detect truly associated genetic loci. Most publicly available control genotype data come from studies with very limited information on PCa status. While there does exist publically available genetic data on PCa screened controls from previous PCa GWA studies, we elected to avoid using controls from these studies in order to obtain independent results. We, and others [36]–[39], have shown that genetic association studies including larger numbers of unscreened controls generally have greater power for discovery than studies using a smaller number of screened controls provided the rate of disease misclassification is not high. For our primary analyses, we chose to include both male and female public controls over a control sample limited to unscreened males. The prevalence of diagnosed PCa in European-American men under 56 years of age is less than 1%, thus the rate of disease misclassification for both our male and female public controls should not be that much larger than it would have been for age-matched screened controls from this age group.

The current study includes a large number of men with positive family history of disease (576/931 had a first or second degree relative with PCa). Some of this enrichment was directly due to ascertainment criteria, but most is likely attributed to increased rates of disease, due to both genetic susceptibility and enhanced screening, in families with early-onset disease. This study adds to the growing evidence that GWA study common variants play an important role in familial and early-onset PCa [17], [25], [40], [41]. As new high-penetrant mutations are detected through next-generation sequencing, assessing the relative role of common risk variants and rare mutations to familial disease clustering will become an exciting area of research. For example, Karlsson et al. [42] recently showed that carrying a *HOXB13* G84E mutation [24], which occurs at a frequency of ∼1.3% in Sweden, is most strongly associated with hereditary (OR = 6.6) and early-onset (OR = 8.6) PCa and that the risk for G84E mutation carriers of developing disease is increased significantly for those carrying a higher burden of established common GWA study variants.

In conclusion, we describe results from the first stage of a two-stage GWA study for early-onset PCa. Our two-stage study design follows the strategy described by Ho and Lange [39], which increases the power of traditional case-control GWA studies by incorporating public control genotype data in the stage 1 discovery phase. As is the case for any study using public control data, care must be taken in interpreting any individual result due to factors such as batch genotyping effects and differential selective pressures across populations, which are difficult to completely control for experimentally or analytically. Our results provide proof of principal that such a study design is reasonable, given the strong evidence at a number of previously established PCa loci and the lack of evidence, with the possible exception of the chromosome 13 rs11839053 finding, for spurious results. In total, our results provide compelling evidence supporting the importance of common genetic variants to early-onset PCa.
